# Experiences of Research Coproduction in Uganda: Comment on "Research Coproduction: An Underused Pathway to Impact"

**DOI:** 10.34172/ijhpm.8806

**Published:** 2024-11-20

**Authors:** David Musoke, Suzan Nakalawa, Michael Obeng Brown, Grace Biyinzika Lubega, Linda Gibson

**Affiliations:** ^1^Department of Disease Control and Environmental Health, School of Public Health, College of Health Sciences, Makerere University, Kampala, Uganda.; ^2^Institute of Health and Allied Professions, School of Social Sciences, Nottingham Trent University, Nottingham, UK.

**Keywords:** Coproduction, Research, Equitable Partnerships, North-South Collaboration, UK, Uganda

## Abstract

This commentary reflects on the principles of research coproduction discussed by Rycroft-Malone et al through our experiences in Uganda, particularly within the partnership between Nottingham Trent University (UK) and Makerere University (Uganda). The commentary highlights the coproduction process we have employed in community health projects in Wakiso district, Uganda, by examining both the opportunities and challenges inherent in this collaborative approach. We further highlight the importance of continuous stakeholder engagement, context-specific communication, and power-sharing, demonstrating how research coproduction can decolonize research methodologies and enhance the relevance and impact of health interventions. By recognising the inequities between North-South partnerships, this commentary contributes to the discourse on how research coproduction can practically be implemented to drive meaningful, community-centred change while addressing the complexities involved. The lessons drawn from our experiences offer a pathway for other global partnerships aiming to integrate the principles of research coproduction into their work.

## Introduction

 Research coproduction is a collaborative approach that can empower researchers and its intended beneficiaries to co-create knowledge, offering immense potential to bridge the gap between research and practice.^1,2^ However, there is an underutilization of this concept in health research globally.^3^ Rycroft-Malone and colleagues highlight this in the editorial “*Research Coproduction: An Underused Pathway to Impact*” by arguing how research coproduction can lead to meaningful change, providing a robust framework for understanding the potential of this approach. This commentary aims to contribute to the ongoing discourse by sharing experiences of research coproduction in Uganda. Particularly, we focus on our work as part of the Nottingham Trent University, UK and Makerere University, Uganda (NTU-Mak) partnership that has implemented several projects among communities in Wakiso district, Uganda for over a decade. By examining the opportunities and challenges encountered in this context, this commentary further enriches the understanding of research coproduction and its implications for achieving meaningful impact.

## NTU-Mak Partnership on Research Coproduction

 This commentary supports the notion that research coproduction can occur at any given stage or level of a project as mentioned in the editorial by Rycroft-Malone and colleagues. We reflect on how we work within a shared ownership model between a high resource and low resource setting. Constant communication at both an informal and formal level with our research community has always been a priority. The stakeholders we engage include community members, community health workers (CHWs), the Wakiso district health team, Ministry of Health (Uganda), and others as dictated by project needs. This regular engagement is specific to the contexts in which our research stakeholders work, depending on their level and preferred mode of communication as highlighted in our book chapter.^4^

 The results of research coproduction amplify the potential for generating evidence-based solutions that can more rapidly translate into better and more equitable health and care, as suggested by the authors. However, as a partnership, we have found that research coproduction is time-consuming and sometimes political due to the dynamics of understanding the different actors, the relations between them, as well as their expectations. This challenge necessitates appreciating that local partners are key to navigating who needs to be involved and their interests in the research and community development. An advantage we have as a partnership is that for nearly 15 years, we have been working in the same district. This has enabled us to understand the different stakeholders and appreciate how best to work with them.^4^ In addition honest conversations, sometimes with disagreement, that require all parties involved to make compromises where necessary have been allowed.^5^ As a partnership, we have come to recognise that the spirit of research coproduction among researchers and knowledge users must first be applied amongst ourselves as a North-South team based in higher education institutions with different access to materials and resources. Recognising these principles enables us to make significant steps to decolonise our research processes, recognising the unequal playing field that exists in the infrastructure and economic contexts that frame our work together especially in the methodologies and interventions we apply.

 Other challenges and tension always exist between North-South collaborations.^4^ We are sensitive to debates about unequal power relations between different socio-economic, cultural and geopolitical regions, as well as active in discussions on decolonising global health and research injustices. The gap in sourcing and managing research funding has meant that we have always taken a mixed funding approach, working with large formal funders and smaller internal strategic grants that fill the gaps. Our partnership addresses publishing injustice by obtaining journal fee waivers for low-income countries and rotating first authorship to promote equity. We always include mobility into our grants to promote knowledge and cultural exchange, but the combination of global economic inequity, pandemics, and energy crises mean that there are different layers of economic hardship. The particularly strained funding for Ugandan students’ accommodation and living expenses in the United Kingdom, alongside delays (and sometimes rejections) in visa issuance has led to additional costs and inconvenience. The length of the partnership and trust have been a major factor in securing senior institutional management support in times of such challenges.^4^

## Community-Based Participatory Research With a Focus on Photovoice

 As an example, we delve into community-based participatory research using photovoice to expound how we utilise principles of research coproduction, giving attention to the voices of marginalized communities. Photovoice is a participatory action research methodology that enables individuals within communities, particularly marginalized groups, to capture and interpret their lived experiences through photography. By granting participants the opportunity to play an active role in the research process, photovoice challenges traditional power dynamics and ensures that their voices are central to knowledge co-creation. We have successfully used this methodology in several studies, focusing on the roles of youth and CHWs in addressing malaria, maternal health, and gender issues.^6-8^ Through visual storytelling, participants constructed and shared their realities using photovoice. The use of photovoice not only generates rich and authentic data but also fosters a sense of agency and ownership among participants, ultimately leading to more relevant, equitable, and impactful research outcomes.

 The concept of research coproduction as discussed by Rycroft-Malone and colleagues aligns closely with the principles underlying the photovoice methodology. The authors discuss how collaborative engagement is integral to the research process, ensuring that the research is significant and impactful.^1^ Similarly, our photovoice studies have enabled participants to have their voices heard and experiences recognised by utilising the photos taken, important principles of coproduction and its ideals of power-sharing and valuing diverse local knowledge. Furthermore, actively involving CHWs in the photovoice studies demonstrates the value of coproduction goals of inclusivity and impact, as emphasized by the authors.^1^ This illustrates how participatory methodologies can translate these principles into tangible, real-world outcomes that empower communities and enrich the research process and outcomes.

## North-South Partnerships, Equity and Facilitating Research Coproduction

 In an increasingly multifaceted and interconnected world, research coproduction can be achievable within well-defined principles and explicit values as noted by the authors.^1^ One distinguishing feature of coproduction is to aim towards a more equitable model of shared ownership between all stakeholders involved in any project especially where there is an existing structured inequity between partnerships such as ours drawn from high and low resource settings.^4^ We concur with the study by Moreno-Cely et al which highlights that to administer a principle-based and values-driven coproduction approach, there is a need to decolonise the research process through continuous building and refining of strategies together with indigenous knowledge holders.^9^ This requires some level of *scientific humility* as highlighted by Hoekstra et al and Bowen.^10,11^

 Within the NTU-Mak partnership, trust is a key distinguishing principle which has underpinned recognising inequities where they exist between us, to be open about them, and work together towards making provision to fill gaps where we can and work with the strengths that exist.^4^ However, there are several challenges researchers may face when attempting to gain the trust of communities and other stakeholders who hold local knowledge. These challenges may often occur because local knowledge holders are at times sceptical about engaging with researchers especially those not from the communities.^12^ Despite challenges around mobility, our partnership has committed to ensuring CHWs and other Ugandan stakeholders including health practitioners have travelled to the United Kingdom as part of ongoing projects. At university strategic and senior management levels, this relationship was strengthened by having a series of memoranda of understanding between us in place, which later enabled Mak to be recognised as one of the four strategic institutional partners of NTU.

 Aside trust, six other principles of the NTU-Mak partnership have emerged in recent years that align with the editorial by Rycroft-Malone et al. These principles include: reciprocity; transparency; cultural appropriateness; global thinking; investment in people; and sustaining activities.^4^ For example, to achieve equity in partnerships, the authors indicate that* mutuality* must be an established theme throughout the research process. This principle is rooted in the NTU-Mak partnership’s principle of *reciprocity* which ensures that our UK partners (such as Buckinghamshire NHS Healthcare Trust) as well as the communities of operation in Uganda (such as health workers at Entebbe Regional Referral and Nakaseke hospitals) mutually benefit from the various interventions, ensuring that learning and knowledge transfer are bi-directional. During these activities, the partnership also ensures that the principle of *open communication* which aligns with that of *transparency* that is upheld by all stakeholders by providing a safe-space to talk freely and willingly.^1,4^ Most importantly, to achieve some measure of equity in partnerships between the North (UK) and South (Uganda), the NTU-Mak partnership team has continuously put the community at the forefront. This has led to the partnership embracing values of equity and social justice, which contributes towards shared ownership throughout our research and other activities.

## Engaging Local Stakeholders to Identify Research Priorities

 Global health research funding bodies remain rooted in biomedical hegemony, and usually are institutionally embedded in high income countries leading to more recent debates about how to decolonise global health both in its structure but also its knowledge base.^13^ The hierarchy of scientific knowledge is often privileged, and true research approaches to coproduction to facilitate knowledge generation are often not highly valued. Our experience in the NTU-Mak partnership aligns with the authors who emphasize the need for current structures, governance and policy frameworks to prioritise indigenous knowledge transfer and dissemination approaches including for research funding bodies. We argue for funders to prioritise coproduction in the different stages of the research process. However, challenging the centre of power relations in coproduction contests the dominant narratives of research and its methodologies.

 From our experience of working with several funding partners, we believe the three levels of engagement (micro, meso and macro) discussed by the authors are crucial. Partnership and “*learning from each other*” have been core principles in working and supporting research partnerships by some funders between high- and low- and middle-income countries. Increasingly, such programmes support the delivery of global health projects that do engage with coproduction and deliver impact and build capacity at various levels. At macro level, some funders are involved in engaging national government ministries including the Ministry of Health and other local stakeholders including non-governmental organisations when conceptualizing funding schemes to identify local research priorities through a series of scoping visits and workshop. At meso level, some funders use narrative reporting templates which have a focus on the use of knowledge by end users. In addition, the funders are keen to have independent conversations with the knowledge end users such as hospitals that are in close contact with patients and communities on any changes as a result of their funding. Just as the authors highlighted, there is a particular gap in the steps to follow to measure the knowledge users’ capability to engage in meaningful research coproduction at the micro level. This is true as knowledge users are not primarily researchers as we have experienced in our partnership work. Nevertheless, funding bodies should allow researchers to explore how best the process of research coproduction with end users can be streamlined and documented for replicability.^14^

## Conclusion

 The experiences of research coproduction in Uganda through the NTU-Mak partnership highlight the immense potential of this collaborative approach in driving meaningful, community-centred change. As Rycroft-Malone et al discussed the benefits of research coproduction such as enhanced relevance, increased impact, and improved relationships and trust, the NTU-Mak partnership has successfully navigated the complexities and challenges inherent in such endeavours over an extended period.^1^ Our commitment to building shared ownership and equitable partnership demonstrates that when research coproduction is applied both within and outside the team, it can foster significant progress toward decolonizing research practices and ensuring that vast voices are respected and heard. As the partnership continues to evolve, we stand as a model for how North-South collaborations can effectively integrate the principles of research coproduction to generate evidence that is not only academically rigorous but also deeply rooted in the needs and aspirations of the communities it serves.

## Ethical issues

 Not applicable.

## Conflicts of interest

 Authors declare that they have no conflicts of interest.
